# First report of changes in leukocyte morphology in response to inflammatory conditions in Asian and African elephants (*Elephas maximus* and *Loxodonta africana*)

**DOI:** 10.1371/journal.pone.0185277

**Published:** 2017-09-21

**Authors:** Nicole I. Stacy, Ramiro Isaza, Ellen Wiedner

**Affiliations:** 1 Department of Large Animal Clinical Sciences, College of Veterinary Medicine, University of Florida, Gainesville, Florida, United States of America; 2 The Wilds, Cumberland, Ohio, United States of America; Universiteit van Amsterdam, NETHERLANDS

## Abstract

Although the hematology of healthy elephants has been well-described, published information on hematological changes during disease is limited. The objective of this study was to describe qualitative morphological changes in the leukocytes of Asian and African elephants (*Elephas maximus* and *Loxodonta africana*) diagnosed with a variety of inflammatory conditions. Twenty-five of 27 elephants had morphological changes in their leukocytes, although only 16 of these had a concurrent inflammatory leukogram. Morphological changes included heterophil left-shifting with or without concurrent dysgranulopoiesis, toxicity, or hypersegmentation, reactive lymphocytes, plasma cells, and/or vacuolated monocytes. Although the observed leukocyte morphological changes are non-specific, their early recognition upon blood film evaluation may provide important, clinically-relevant information, particularly if the leukogram is normal. This case series is the first description of qualitative morphological changes in the leukocytes of elephants in association with inflammation.

## Introduction

The complete blood count (CBC) is an essential part of the minimum baseline diagnostics in Asian (*Elephas maximus*) and African (*Loxodonta africana*) elephants managed in human care. Both hematological reference intervals and normal leukocyte morphology of healthy elephants have been reported. Elephants, like other members of the *Afrotheria*, especially the well-studied hyraxes and manatees possess leukocytes with several unique features. These include having heterophils instead of neutrophils and a unique monocyte type with a bilobed—but sometimes tri-lobed—nucleus and peroxidase-positive cytoplasmic staining. This unique monocyte is observed in addition to the typical monocyte type of other mammals [1,2,3]. Both types are grouped together as monocytes during white blood cell (WBC) differential counts. In fact, monocytes represent the most abundant leukocyte type in elephants [1,2].

Elephant eosinophils, basophils, and lymphocytes are readily identifiable upon blood film evaluation since they are morphologically very similar to those of other mammalian species. Basophils, however, are rarely observed in either elephant species [2]. A challenge in elephant hematology is the proper differentiation of lymphocytes from the unusual monocyte type described above. Confusion in identifying these two cell types has resulted in higher reported absolute lymphocyte numbers in the older literature [2], and caution has been recommended in using previously published reference intervals since large standard deviations in lymphocyte and monocyte counts likely reflect inconsistency in cell differentiation [2]. This differentiation of elephant lymphocytes and monocytes is very challenging for blood film evaluation, and no information exists about the accuracy of hematology analyzers. Correct cell differentiation, however, is the basis for accurate leukocyte differential counts and recognition of changes in absolute numbers of WBC in the CBC of an individual patient.

Surprisingly, descriptions of detailed morphological features of elephant leukocytes in response to disease are lacking in the literature, since abnormal morphology is obviously absent in healthy elephants [2]. This is the first report describing qualitative morphological changes in leukocytes in a case series of elephants with various inflammatory conditions.

## Material and methods

A clinical pathology database was reviewed to identify elephant samples linked with various inflammatory conditions. Elephant samples came from multiple elephant-holding facilities located in the United States. All blood samples were collected under due consideration of the responsible veterinarian’s discretion. The database contained CBC results generated from whole blood samples collected at each elephant’s home facility. In detail, blood samples were preserved in ethylenediaminetetraacetic acid (EDTA)-coated vacuum tubes from which two blood films were prepared within one to two hr of blood collection. The blood films were air-dried, placed in cardboard or plastic slide containers, then shipped overnight to a clinical pathology laboratory along with the EDTA-preserved blood tubes on cold packs. Upon arrival at the lab, samples were kept at refrigerated temperatures (between 0 and 4°C) until processing within two hours of arrival. For comparative purposes, in a few cases, an additional set of blood films was made at the laboratory, more than 24 hr after the blood was drawn. An automated analyzer (Advia® 120, Siemens Medical Solutions Inc., Malvern, Pennsylvania 19355 USA) performed a routine CBC on each sample including a WBC count. The WBC count from the analyzer was used for the calculation of absolute WBC numbers based on a 200 WBC differential by blood film evaluation. An inflammatory leukogram was defined as a WBC count greater than >13.6 K/μl, a heterophil bands greater than 0 K/μl, segmented heterophils greater than 3.2 K/μl, lymphocytes greater than 2.7 K/μl, and/or monocytes greater than 7.1 K/μl. These numbers are the mean from previously published reference intervals for captive elephants [2].

All blood films were stained with Wright–Giemsa (Harleco®, EMD Millipore, Billerica, Massachusetts USA 01862) prior to a full blood film evaluation [4]. The WBC morphology was carefully evaluated using a 100x microscope objective.

## Results

Twenty-seven elephants, consisting of 16 females and 11 males, were identified with the following clinical diagnoses: non-specific gastrointestinal disease (n = 12), salmonellosis (n = 2), chronic foot disorders (n = 5), elephant endotheliotropic herpesvirus (EEHV) infection (n = 4), dental issues (n = 2), a retained fetus causing metritis (n = 1), and metastatic ovarian adenocarcinoma (n = 1). Twenty-two of these elephants had multiple samples submitted during the course of their disease. Elephant ages ranged from 5 mo to 63 yr. Elephants included 17 Asian and 10 African elephants. Twenty-four elephants fully recovered from their clinical conditions. Three elephants died of their medical problems and included an African elephant that developed metritis then sepsis due to a retained fetus, an Asian elephant with metastatic ovarian adenocarcinoma, and an Asian elephant with EEHV (Table 1).

**Table 1:** Overview of qualitative leukocyte morphological changes observed in blood films at initial sampling from 27 elephants with inflammatory conditions.

Species	Clinical condition	Inflam-matory leukogram Y/N	Out-come	Age (yr)	Sex	Band H	Heterophil left-shifting back to myelocytes	Dys-granulo-poiesis	Heterophil toxicity	Heterophil hypersegmen-tation	Reactive lympho-cytes	Plasma cells	Activated monocytes
Em	GID	Y	REC	21	F	x	-	-	x	-	x	-	x
Em	GID	Y	REC	53	F	-	-	-	-	-	x	-	-
Em	GID	N	REC	9	F	x	-	-	-	-	x	-	x
Em	GID	N	REC	51	M	-	-	-	-	-	x	-	x
Em	GID	N	REC	12	F	x	-	-	-	-	-	-	x
Em	GID	Y	REC	35	M	-	-	-	x	-	x	x	-
Em	GID	Y	REC	29	F	x	x	-	x	-	-	-	x
Em	CFD	N	REC	41	M	x	-	-	-	-	x	-	-
Em	CFD	N	REC	51	F	x	-	-	-	-	-	-	x
Em	CFD	Y	REC	45	M	-	-	-	-	-	-	-	-
Em	CFD	Y	REC	36	F	-	-	-	-	-	-	-	-
Em	CFD	N	REC	59	F	-	-	-	-	-	x	-	x
Em	EHV	Y	DIED	6	M	x	x	x	x	-	x	-	x
Em	EHV	Y	REC	0.5	F	x	x	x	-	-	x	-	x
Em	EHV	Y	REC	9	M	x	x	-	x	-	-	-	x
Em	EHV	Y	REC	4	M	x	-	-	x	-	x	-	-
Em	ACA	N	DIED	NA	F	x	-	-	x	-	-	-	x
La	GID	Y	REC	17	F	x	-	-	-	-	x	-	-
La	GID	Y	REC	23	F	-	-	-	-	x	-	-	x
La	GID	Y	REC	27	M	x	x	x	x	-	-	x	x
La	GID	N	REC	11	F	-	x	x	x	-	x	-	x
La	GID	N	REC	38	M	x	-	-	x	-	-	-	x
La	SAL	Y	REC	21	F	x	-	-	-	-	x	-	-
La	SAL	N	REC	42	M	x	-	-	x	-	-	-	x
La	DEN	Y	REC	63	M	-	-	-	-	-	-	-	x
La	DEN	N	REC	54	F	x	-	-	x	-	x	-	x
La	RFE	Y	DIED	NA	F	x	-	-	x	-	-	-	x

Legend: Em = *Elephas maximus*; La = *Loxodonta africana*; H = heterophil; GID = gastrointestinal disease; SAL = salmonellosis; CFD = chronic foot disorders; EHV = elephant endotheliotropic herpesvirus infection; DEN = dental issues; RFE = retained fetus; ACA = metastatic ovarian adenocarcinoma; REC = recovered. NA = Not available.

**Fig 1:**
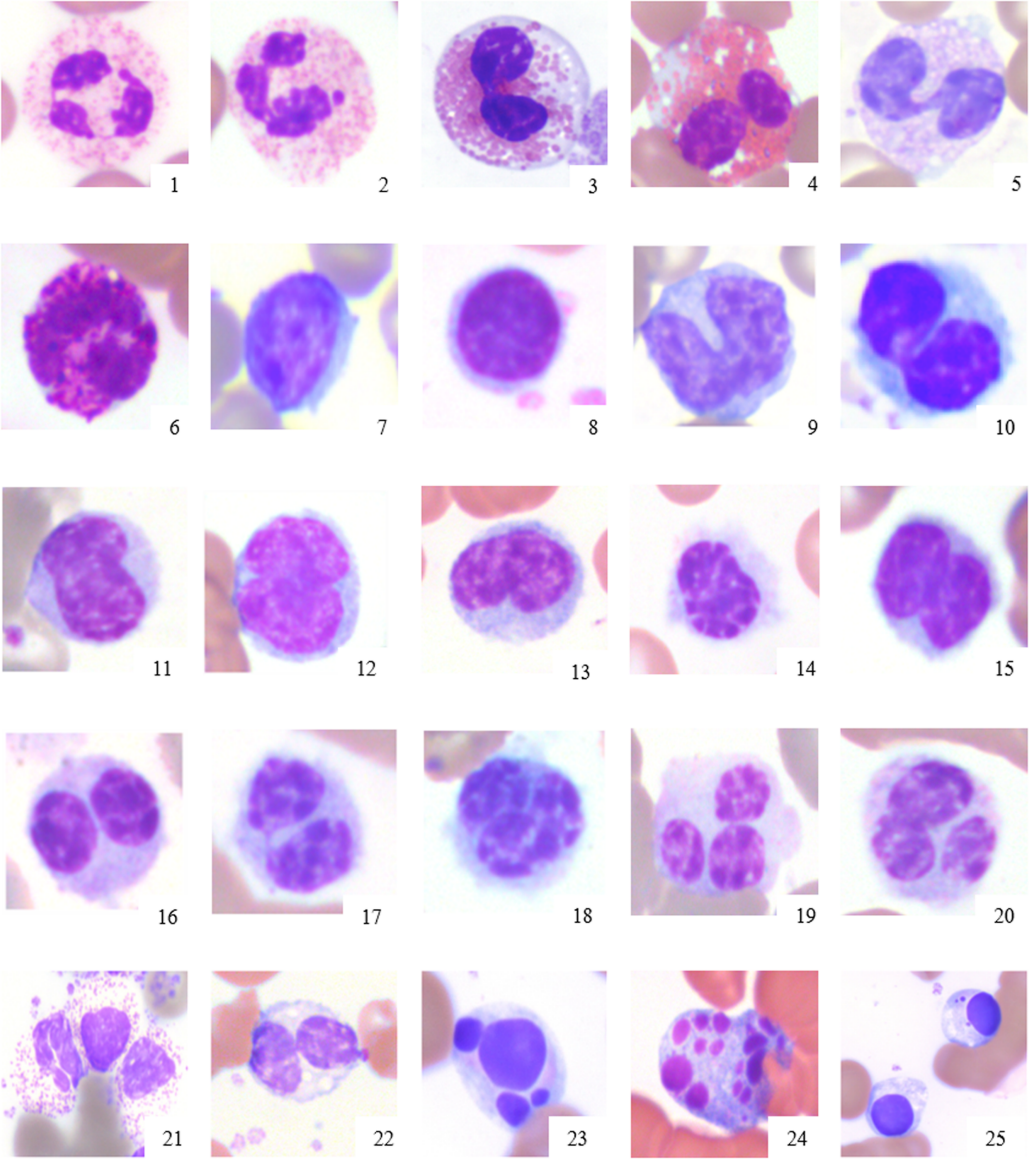
Normal morphology of elephant leukocytes. 1: Mature heterophil, 2: Mature heterophil with Barr body in a female elephant (sex chromatin lobe), 3–4: eosinophils, 5–6: basophils, 7–8: small lymphocytes, 9: monocyte, 10–20: unique monocyte type of elephants with various stages of segmentation; note that less segmented monocytes may be misidentified as lymphocytes. 21–25: degenerative changes in leukocytes in blood films prepared 24 hr after collection, including 21: heterophil with nuclear and cytoplasmic swelling, 22: monocyte with swollen nucleus, 23–25: karyorrhexis and pyknosis in leukocytes. X100 objective. Wright Giemsa stain.

**Fig 2:**
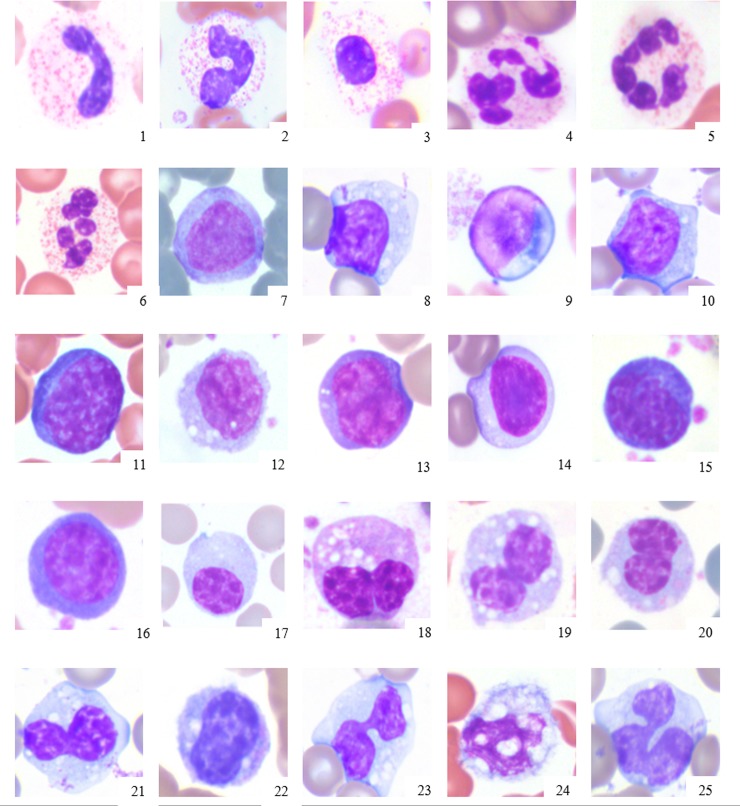
Abnormal morphology of elephant leukocytes. Legend: 1–2: Band heterophils, 3: heterophilic myelocyte, 4–6: hypersegmented heterophils, 7–16: reactive lymphocytes, 17: plasma cell, 18–25: activated monocytes with variable vacuolation. X100 objective. Wright Giemsa stain.

Twenty-five of the 27 elephants had morphological changes in their leukocytes. Two adult Asian elephants with mild chronic foot disorders did not have morphological changes in their leukocytes although both had an inflammatory leukogram characterized by a mild monocytosis. In elephants with leukocyte morphological changes, one or more of the following were observed: heterophil left-shifting with or without concurrent dysgranulopoiesis, heterophil toxicity, hypersegmented heterophils, reactive lymphocytes, presence of plasma cells, and vacuolated/activated monocytes (Figs 1 and 2). The described morphological leukocyte changes were observed in both the presence (n = 14) and absence (n = 11) of an inflammatory leukogram.

**Fig 3:**
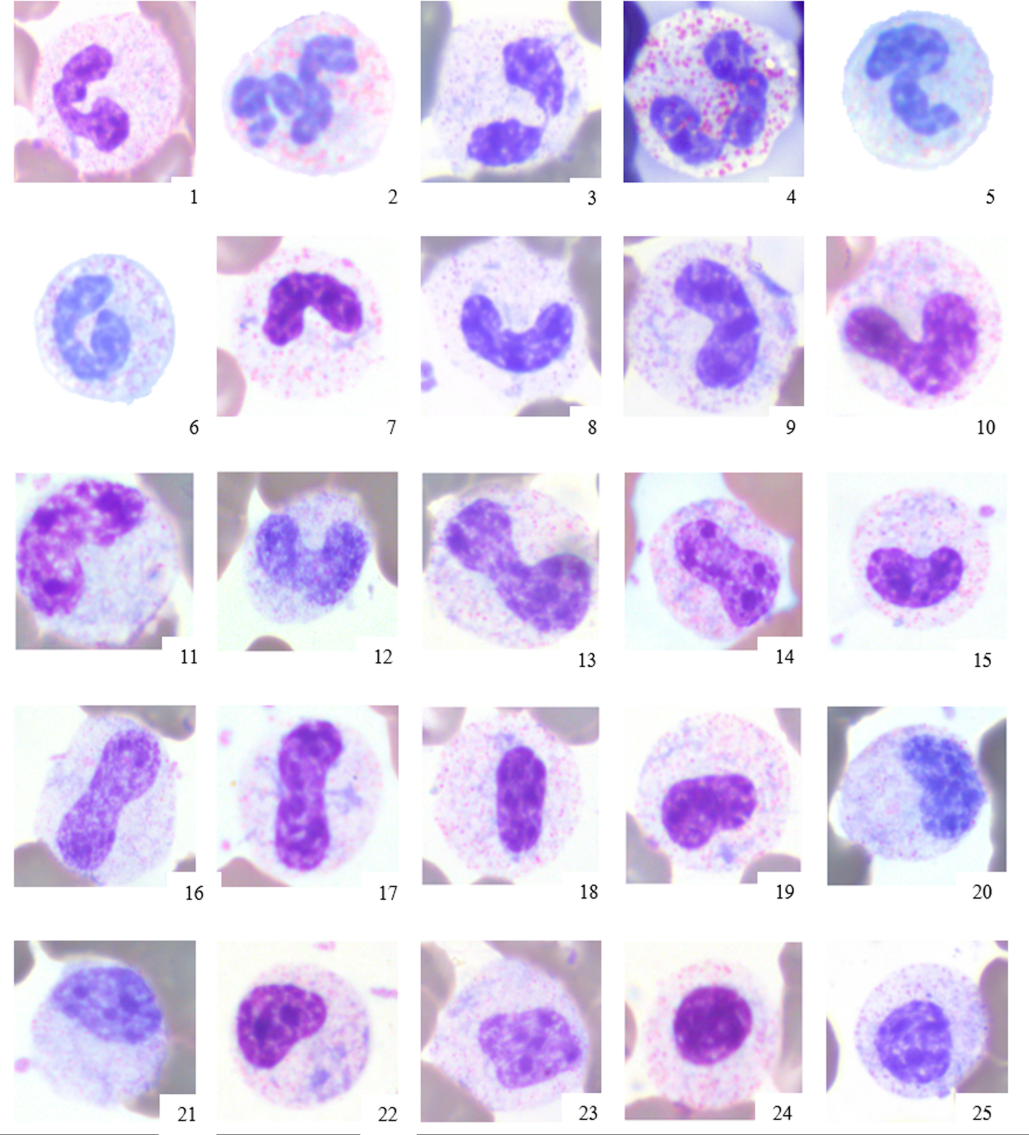
Heterophil left-shifting and variable toxicity in elephant blood films. 1–6: Mature heterophils, 7–12: band heterophils, 13–20: heterophilic metamyelocytes, 21–25: heterophilic myelocytes. Grading of toxicity on a scale of 1+ (Doehle bodies, slight basophilia), 2+ (Doehle bodies, moderate basophilia), and 3+ (cytoplasmic foaminess (dark blue cytoplasm, Doehle bodies may or may not be visible) [4]. 1+ toxicity is present in 1, 7; 2+ toxicity is present in 2, 3, 8, 10, 13–15, 17–19, 22–24; 3+ toxicity is present in 4–6, 9, 11–12, 16, 20–21, 25. X100 objective. Wright Giemsa stain.

**Fig 4:**
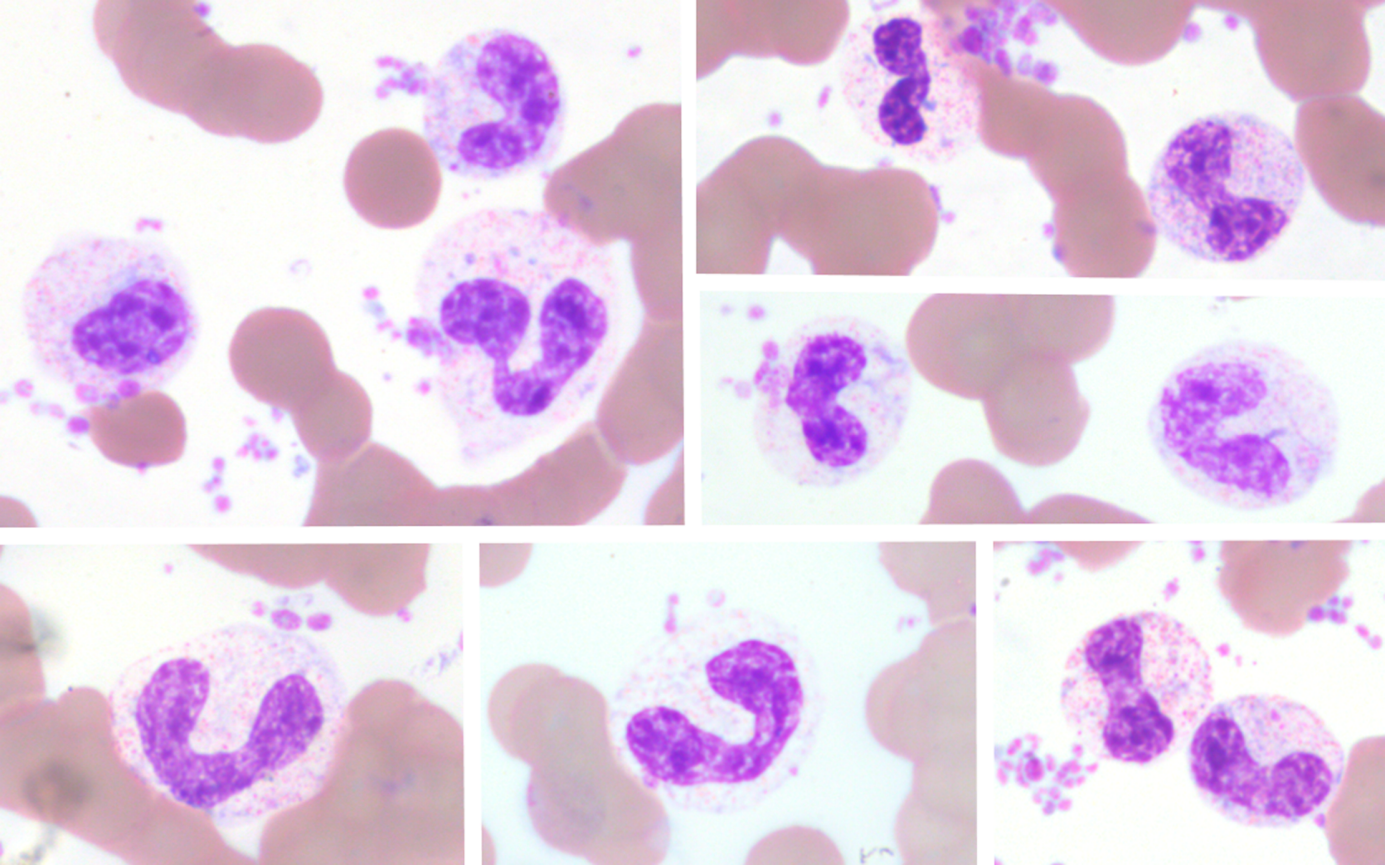
Heterophil left-shifting and variably toxicity in an Asian elephant with gastrointestinal disease for demonstration of the size variation from concurrent secondary dysgranulopoiesis (giant cells). X100 objective. Wright Giemsa stain.

The most frequently observed leukocyte change was heterophil left-shifting. Heterophil left-shifting was characterized in most cases by the presence of band heterophils. Five elephants exhibited left-shifting that also included heterophil metamyelocytes and myelocytes, accompanied in four elephants by secondary dysgranulopoiesis (i.e., abnormal heterophil precursor maturation and/or morphology) characterized by large heterophils. Earlier heterophil precursors such as promyelocytes or myeloblasts were not present in any cases. Heterophil left-shifting often occurred concurrently with toxicity, which was characterized by the presence of Doehle bodies and variable cytoplasmic basophilia on a scale of 1+ to 3+ per standard hematology practice [4]. (Figs 3 and 4) Toxicity developed most frequently in this order, starting with Doehle bodies and increasing in severity of cytoplasmic basophilia in conjunction with worsening disease. Hypersegmented heterophils characterized by five or more lobes without any visible cytoplasmic toxicity were noted in one African elephant with chronic inflammation due to gastrointestinal disease.

Reactive lymphocytes were identified based on the presence of increased amounts of cytoplasm, increased cytoplasmic basophilia, and/or increases in cell size. Plasma cells were present in two elephants and were similar in morphology as described in other species. Activated monocytes were characterized by the presence of variable degrees of vacuolation. Rare trinucleated macrophages were also observed.

At time of recovery, 22 elephants with initial WBC changes no longer had leukocyte morphological changes in their follow-up blood film evaluations. In contrast, the three elephants that died of their medical problems exhibited increasingly severe heterophil toxicity and left-shifting as their conditions progressed. In the two elephants with chronic foot problems that did not have leukocyte changes initially, no morphological changes were noted in follow-up blood evaluations either.

Leukocytes showed several morphological changes after 24 hr of storage that included cell and/or nuclear swelling, cytoplasmic vacuolation, karyorrhexis, and/or pyknosis. Heterophil cell swelling could be clearly identified as storage artifact if it occurred alone and without toxic cytoplasmic changes.

## Discussion

This is the first description of qualitative morphological changes in Asian and African elephant leukocytes associated with various inflammatory conditions. The observed morphological leukocyte changes were non-specific for the underlying conditions and there were no obvious species differences in response to inflammation. Leukogram variations in an individual animal, especially subtle changes, require accurate identification of cells to be made during the WBC differential by blood film evaluation. This is especially important with regard to the differentiation of elephant monocytes and lymphocytes, and correct identification of left-shifted heterophils. Quantitative leukogram changes provide important clinical information and can occur with various infectious diseases. In several reports, elephant endotheliotropic herpesvirus (EEHV) viremia in Asian elephants correlated with significant leukopenia and thrombocytopenia, whereas resolution of viremia was associated with leukocytosis and thrombocytosis [5,6]. Other hemogram alterations that have been seen in clinical cases of EEHV include monocytopenia, monocytosis lymphocytosis, lymphopenia, and anemia, but these may be very variable during the course of disease [7,8]. Band heterophils and increased numbers of eosinophils and platelets were reported in Asian elephants that were culture-positive for *Mycobacterium tuberculosis* [9].

As this descriptive case series shows, even elephants with normal leukograms may have morphological changes in their leukocytes indicative of inflammation. Although low numbers of band neutrophils are considered normal in other mammalian species, band heterophils or cytoplasmic toxic changes in heterophils should not be present in blood films from healthy Asian or African elephants [1,2]. Left-shifting in mammals, i.e., an increased number of immature neutrophils, most frequently is a non-specific indicator of inflammatory disease from either infectious or noninfectious causes [4]. In the elephant cases described here, left-shifting back to the myelocyte stage was observed but not to the most immature stages, which is similar to other mammals [4]. The morphology of left-shifting was identical to that described in other mammals [4]. The presence of giant heterophils in elephants with concurrent left-shifting is consistent with secondary dysgranulopoisis resulting from shortened bone marrow maturation time and thus premature release of precursor cells in response to peripheral demand [4]. Left-shifting may often be present in conjunction with toxicity which although non-specific, may be associated with inflammation due to release of immature stages of neutrophils/heterophils, infections (e.g., bacterial, viral), and/or metabolic derangements [4]. Similar to other mammals, the elephants’ degree of heterophil toxicity appears to correlate with the severity of clinical disease [8], and heterophil toxicity resolved with clinical improvement but worsened with disease progression. Hypersegmentation, seen in one elephant with gastrointestinal disease, was likely associated with chronic inflammation [4]. Other causes of hypersegmentation, such as delay in processing or glucocorticoid administration, were not applicable to this elephant patient.

Abnormal lymphocyte morphology observed in elephants was very similar to typical morphological changes described in other mammals with inflammation. A reactive lymphoid population is characterized by a heterogenous mixture of lymphocytes exhibiting morphological features such as variation in size, increased cytoplasmic basophilia, and/or increased amount of cytoplasm [4].

In contrast to reactive lymphocytes, activated monocytes are defined by the presence of variable numbers of cytoplasmic vacuoles. Since the unique bilobed elephant monocyte may present in various stages of maturation, i.e. slight indentation of a round to oval nucleus, it is necessary to emphasize the importance of the proper differentiation of lymphocytes and monocytes as a basis for an accurate WBC differential.

While very low numbers of reactive lymphocytes and/or activated monocytes in healthy animals of other mammalian species are considered normal [4], their presence in elephants should be considered abnormal as they have not been documented in healthy animals [1,2,3]. The presence of reactive lymphocytes, plasma cells, or activated monocytes in blood films from various domestic mammals has been associated with antigenic stimulation from various causes [4]. Their presence in an elephant blood film warrants consideration of further relevant diagnostic testing. The rarely observed trinucleation of monocytes was considered a morphological variant of the binuclated monocyte type. Since the authors have observed trinucleated monocytes in both healthy and non-healthy elephants, their clinical significance is unknown at this time.

Why two elephants with chronic foot problems did not have changes in their leukocyte morphology remains unclear, although both had a mild monocytosis. Possibly some types of foot problems, especially those that are mild and chronic as in these two elephants, eventually cease to cause a significant enough level of an inflammatory response to result in leukocyte morphological changes. Inflammation, in general, refers to a series of responses made by the immune system in response to multiple stimuli that may include microorganisms, trauma, radiation, or chemicals [4,10]. Although some aspects of the elephant inflammatory response have been explored, including acute phase proteins [11,12] and cytokines [13,14], the full spectrum of triggers, components, and responses remains, for the most part, unknown. Understanding of these issues may eventually explain why the quantitative elephant leukogram (i.e. changes in absolute numbers of WBC types) often does not change until late in disease, a phenomenon shared with their close relatives, the manatees [15].

The morphological changes noted in blood films prepared 24 hr after blood draw emphasizes the importance of timeliness in making blood films. Morphological changes due to artifact can resemble those resulting from mild toxicity (e.g., vacuolation) or may hinder proper cell identification. The degree of change may reflect the amount of elapsed time between blood draw and blood film manufacture or temperature variations during shipment to a laboratory. Although the time course of such changes is unknown, delays in processing should be noted before interpreting toxic changes on a blood film, and facilities should strive to make blood films as soon as possible after blood collection for best sample quality.

This report highlights the importance of blood film evaluation as part of the CBC for routine wellness examinations as well as for diagnosing sick elephant patients. Quantitative leukogram changes may be absent in sick elephants until advanced stages of disease. Thus, evaluation of qualitative changes in appropriately prepared blood films using standard methods of evaluation, which include describing the severity of toxicity on a scale of 1+ to 3+ and providing a subjective estimate of affected heterophils (e.g. few, occasional, most, all) [4] may yield valuable clinical information earlier in the disease process and for monitoring of the patient. Given the potential for discrepancies in the differentiation of lymphocytes and monocytes by hematology analyzers as well as by blood film evaluation, the authors consider the manual WBC differential superior over the automated analyzer differential, since a detailed morphologic evaluation can be accomplished for the trained professional when the unique morphological features of elephant monocytes are considered.

Fresh blood films can also be helpful in monitoring patients during the course of disease as evidenced by the majority of elephants in which leukocyte abnormalities disappeared after recovery and by the three elephants whose heterophil toxicity worsened as their conditions declined. In EEHV cases, confirmation of diagnosis requires at least 24 hr for completion of PCR, and measurements of viral loads are not always possible. Additionally, some infected young elephants show variable and vague signs that confound even recognition of whether they are actually sick [16,17]. In these cases, the appearance of band heterophils in the blood smear of a juvenile Asian elephant should raise a high level of clinical suspicion of significant systemic inflammation, and EEHV should be considered as a differential. This descriptive case series provides the first documentation of morphological changes of leukocytes in elephants with inflammatory conditions, and offers an important, early diagnostic tool to clinicians working with elephants.

## Conclusions

The leukocytes of elephants affected by various inflammatory conditions can exhibit subtle to significant morphological changes with or without an abnormal leukogram. The proper recognition of these morphological changes by blood film evalution provides clinically-relevant diagnostic information early in the disease and is useful for monitoring patient progress. Blood films prepared within one to two hrs of collection provide optimal quality to detect morphological changes in elephant leukocytes.
